# Evidence That Baseline Levels of Low-Density Lipoproteins Cholesterol Affect the Clinical Response of Graves’ Ophthalmopathy to Parenteral Corticosteroids

**DOI:** 10.3389/fendo.2020.609895

**Published:** 2020-12-22

**Authors:** Adriano Naselli, Diletta Moretti, Concetto Regalbuto, Maria Luisa Arpi, Fabrizio Lo Giudice, Francesco Frasca, Antonino Belfiore, Rosario Le Moli

**Affiliations:** Endocrinology, Department of Clinical and Experimental Medicine, University of Catania, Garibaldi-Nesima Medical Center, Catania, Italy

**Keywords:** cholesterol, low-density lipoprotein cholesterol, Graves’ ophthalmopathy, parenteral corticosteroids, Graves’ disease

## Abstract

**Background:**

High dose intravenous glucocorticoid (ivGC) therapy is the first line treatment in moderate to severe Graves’ ophthalmopathy (GO) and is associated with a clinical response rate ranging from 50% to 80%. Recently, a positive correlation between total cholesterol and low-density lipoproteins cholesterol (LDLc) with GO presentation and activity has been described.

**Objective:**

We aimed at evaluating whether, in patients with moderate to severe active GO treated with ivGC therapy, cholesterol, and LDLc could represent valuable predictive factors of medium-term GO outcome.

**Methods:**

This single center retrospective study was conducted in a consecutive series of 87 patients undergone ivGC therapy because affected by moderate to severe active GO. Clinical outcome of GO was evaluated at week 6 (W6) and 12 (W12) in respect to baseline conditions (week 0) by the seven points CAS according to EUGOGO recommendations. Univariate analysis and binary logistic regression were performed for the outcome variable W12CAS.

**Results:**

In patients with active GO, an early positive clinical response to ivGC therapy (as evaluated by CAS at 6W) was a strong determinant (OR=13) of the clinical outcome at week 12. Moreover, high levels of LDLc at baseline were positively associated with a reduction in the likelihood of being classified as improved at 12W. Patients with LDLc >193.6 mg/dl were very likely to respond negatively to ivGC therapy independently from the response at 6W. Based on these results, we propose a predictive decision-making model to be tested in future prospective studies.

**Discussion:**

We found that, in patients with active GO, both an early clinical response to ivGC therapy and baseline LDLc levels are significant determinants of GO outcome (W12CAS). These data support the need of a cholesterol-lowering treatment before addressing these patients to ivGC therapy.

## Introduction

Graves’ disease (GD) is an autoimmune disorder associated with the production of activating autoantibodies to the thyroid-stimulating hormone receptor (TSH-R) in the thyroid gland, leading to hyperthyroidism. A major extra-thyroidal complication of GD is Graves’ ophthalmopathy (GO), an autoimmune and inflammatory condition characterized by orbital disfigurement, double vision, and decreased visual performance up to blindness (sight-threatening GO). All these factors are associated with proven decrease of quality of life and negative social impact (1, 2). Innate and adaptive immunity as well as a large number of inflammatory factors are implicated in GO pathogenesis (3). High dose ivGC therapy is the first choice in moderate to severe or severe active GO; other immunosuppressive drugs and/or retrobulbar irradiation are widely used in the treatment of GO. Surgical orbital decompression is, however, the best choice for treating patients with moderate to severe or severe inactive GO. Treatment with 131-iodine, severe hyperthyroidism and elevated anti TSH-R antibodies (TRAbs) are considered risks factors for GO presentation or exacerbation (4). Smoking is considered to be the most important risk factor for presentation, exacerbation of GO and resistance to corticosteroids (5). Low levels of miR-224-5p also appear to reduce the clinical efficacy of medium dose of ivGC therapy in patients with moderate to severe active GO (6). Recently a retrospective analysis of a large number of patients showed an independent effect of 3-hydrossi-3methylglutaryl CoA reductase inhibitors (statins) on the risk to develop GO (7). In patients with GD, statin use for approximately 60 days during a one-year observation period significantly reduced the risk of developing GO, an effect not obtained when patients were treated with cyclooxygenase-2 (COX-2) inhibitors alone (8). A significant correlation of GO presentation and activity with total cholesterol and LDLc has been described (9) revealing a role of cholesterol in the processes involved in GO pathogenesis. Those data suggest the possibility that cholesterol might not only increase the risk of GO but also its activity, corroborating previous data showing that chronically elevated cholesterol increases systemic inflammation and modulates innate and adaptive immunity (10). However, the possible impact of cholesterol levels on the clinical efficacy of ivGC therapy in patients with active GO is not known (8, 11, 12).

We aimed at evaluating whether serum cholesterol levels may serve as predictive factor of medium-term clinical activity GO outcome in patients with moderate to severe active GO treated with ivGC therapy.

## Patients and Methods

### Patients

We retrospectively selected 87 consecutive patients with Graves’ disease and GO referred to our out-patients thyroid clinic from January 2013 to July 2019 and treated with ivGC therapy because of moderate to severe active GO. Patients with a history of treatment with statins, corticosteroids, radioiodine (RAI) for Graves’ hyperthyroidism, retrobulbar radiotherapy or with incomplete metabolic evaluation were excluded from the study. Six patients previously treated with thyroidectomy 11.1 (14.6) months earlier and stably euthyroid were included.

### Graves’ Ophthalmopathy Clinical Evaluation at Baseline

All patients were evaluated at the combined thyroid eye clinic (CTEC) of Endocrinology Unit at Garibaldi-Nesima Hospital, Catania, Italy. The same expert endocrinologist carried out the clinical examination of GO according to European Group of Graves’ Ophthalmopathy (EuGoGo) criteria (4). The lid fissure width was evaluated in millimeters by a router, proptosis of each patient was evaluated by the same Hertel exophthalmometer. Diplopia was classified according to the Gorman score by four levels of severity: absent, intermittent, inconstant, or constant. A complete ophthalmological evaluation was carried out by the same expert ophthalmologist. The GO was defined as moderate to severe when eye disease had a sufficient impact on daily life with one or more of the following clinical signs, each part of a composite index (CI): lid retraction 2 mm or more, moderate or severe soft tissue involvement, exophthalmos 3 or more mm above 21, and inconstant or constant diplopia. GO activity was evaluated according to seven points CAS and was considered active when reaching a CAS ≥3.

### Graves’ Ophthalmopathy Clinical Outcome Evaluation

Clinical outcome of GO was evaluated at week 6 (W6) and 12 (W12) in respect to baseline conditions (week 0) by the seven points CAS and was defined clinically improved when CAS improved by almost 2 points in at least one eye. Deterioration was defined by CAS worsening of at least 2 points or when dysthyroid optic neuropathy (DON) or corneal breakdown occurred. No changes were defined as the condition in which changes less than those described above occurred. For each assessment, patients who improved were considered as group I, patients who did not improve or who worsened were defined as group NI. According to these groups and to the time of assessment we defined the following categories: week 6 CAS (I_W6CAS_ or NI_W6CAS_), week 12 CAS (I_W12CAS_ or NI_W12CAS_).

### Pulse Therapy

Corticosteroid treatment for GO consisted of ivGC (Solumedrol; Pfizer, Karlsruhe, Germany) injections with a median cumulative dose of 52.3 mg/kg subdivided in 12 weekly infusions. Patients were asked to report to our endocrinology day hospital on the appointed day, an indwelling venous catheter was inserted into an antecubital vein between 8.30 and 9.30 and Solumedrol diluted in 250 ml of a 0.9 sodium chloride solution % was administered at an infusion rate of 120 ml/h in the post-absorption state and in a lying position. Blood pressure, blood glucose, lipid levels, thyroid, liver and kidney function were evaluated prior to initiating the corticosteroid infusion.

### Analytical Methods

Serum hormones were measured by microparticle enzyme immunoassay (Abbot AxSYM-MEIA) with inter-assay coefficients of variation of less than 10% over the analytical ranges of 1.7–46.0 pmol/L for FT3, 5.15–77.0 pmol/L for FT4, and 0.03–10.0 mU/L for TSH. The within-run and between-run precisions for FT3, FT4, and TSH assays showed coefficients of variation <5%. Thyrotropin receptor antibodies (TRAbs) were measured by a III generation assay (SELco TRAbs Human, Dahlewitz/Berlino (Germany). Glycaemia, total cholesterol, high density lipoprotein (HDL) cholesterol and triglycerides were evaluated by standard methods. We calculated LDLc values by the formula of Martin/Hopkins that is reliable for a wide range of triglyceride values (13). The thyroid was evaluated by ultrasound and the volume of each lobe was calculated with the formula for ellipsoid volumes: Volume = length*width*depth*(π/6). The calculated thyroid volume was the sum of the volumes of the two lobes (14).

### Statistical Methods

Statistical analyses were performed with the SPSS package (IBM SPSS Statistics for Windows, Version 26.0. Armonk, NY: IBM Corp). For the descriptive analysis, continuous variables were expressed as median (25th-75th percentile); categorical variables were expressed as numbers and percentages. We calculated that, to detect a moderate size effect (OR = 2) with a p value ≤ 0.05 and a statistical power of 0.8, a total of 84 patients would have needed (see supplementary data).

#### Univariate Analysis

Univariate analysis was performed to identify predictive variables significantly associated with the clinical outcome at W12CAS (“Improved” and “Not Improved”). Continuous variables were analyzed using the non-parametric Mann Whitney U test. The shapes of distribution of each variable were evaluated by visual inspection of the population pyramid charts; for distributions of similar shape we reported the medians, for distributions of different shapes we reported average ranks. The estimate of the effect size “r” was calculated when appropriate (15). Categorical variables were analyzed by the Chi-square test or, if cells with less than five expected cell numbers were found, by Fisher’s exact test. The strength of the associations was quantified by calculating the Odds Ratio.

#### Binary Logistic Regression

A binary logistic regression was performed for the outcome variable (W12CAS). Covariates were selected on the basis of the results of univariate analysis and the final model was built using forced entry and a hierarchical method. Linearity of the continuous variables with respect to the logit of the dependent variable was assessed by the Box-Tidwell procedure (16) and a Bonferroni correction was applied using all terms in the model to assess its statistical significance (17). Multi-collinearity was excluded after checking tolerance (18) and variance inflation factor (VIF) (19) statistics and the proportion of the variance of each predictor’s b value attributed to each eigenvalue.

The possible presence of outliers was verified by examining standardized residuals and values ≥1.96 or ≤-1.96 standard deviations were reported; cases whose standardized residuals exceeded such thresholds were inspected closely and the decision to eliminate or to keep them in the analysis was made after assessing if they exerted an undue influence on the model using the following influence statistics: Cook’s distance, DFBeta, and Leverage statistics. The adequacy of the models was tested with the maximum likelihood method. The Wald test was used to verify that coefficients differed from 0. Odds ratios were calculated as the exponential of b values to give an indicator of the change in odds resulting from a unit change in the predictor. The proportion of variation in the dependent variable explained by each model was assessed by Nagelkerke pseudo-R^2^ value (20). To predict the probability of improving at week 12 (W12CAS), we constructed our models on the basis of the theoretical model of the binary logistic regression, expressed by the following equation [Eq. 1]:

(1) $$P(Y)=\frac{1}{1+e-\left(b_{0}+\sum_{i=1}^{n}b_{i}x_{i}\right)}$$

Where:

*P (Y)*: probability of *Y* occurring; in our study *Y* = I_W12CAS_.

*e:* Euler’s number

*b_0_*: constant regression coefficient (interception at x axis)

*b_i_*: regression coefficient of *x_i_*

*x_i_*: predictor variable

*n*: number of predictor variables

Logistic regression models assigned a predicted category on the basis of P (Y) values (predicted probability of improving). Using the standard cut-off P (Y) = 0.5 the models assigned a predicted category = Improved (I_W12CAS_) when P (Y) ≥ 0.5; the remaining predicted category (NI_W12CAS_) was assigned if P (Y) was < 0.5.

#### Receiver Operating Characteristic Curve Analysis and Youden’s Test

These analyses were performed for the W12CAS binomial logistic regression model on the basis of the regression outputs. The ability of the model to discriminate between outcome categories was investigated in more details by elaborating the ROC curve (21). The P(I_W12CAS_) (predicted probability of improving at W12) provided by regression analysis was set as test variable, in order to study the model accuracy for different P(I_W12CAS_) cut-offs. The cut-off with the best compromise between sensitivity and specificity was assessed using Youden’s test (22).

All tests were considered statistically significant at a 2-tailed p value <0.05.

## Results

Patient characteristics at study entry are shown in **Table 1**. Eighty-seven patients completed the 12-week (12W) GO clinical assessment according to the seven-points CAS and 71.3% of them improved. Fifty-three patients who had a complete metabolic assessment were included in the analysis.

**Table 1:** Characteristics of patients at study entrance.

Variables			
Number of patients		87	
ivGC treatment period		1/2013–7/2019	
Sex: male/female (n, %)		22 (25.3)/ 65 (74.7)	
Age (years)		45 (35–55)	
BMI (Kg/mq)		24.1 (21.6–28)	
Current smokers (n, %)		45 (51.7)	
Previous Smokers (n, %)		29 (33.3)	
Non Smokers (n, %)		13(14.9)	
Thyroid volume (ml)		18.1 (13–24.3)	
ivGC cumulative dose (mg/kg)		52.3 (40.2–68.2)	
CAS		3 (3–4)	
GO duration (months)		8 (3–12)	
TSH (mU/L)		0.05 (0–0.9)	
FT3 (pg/ml)		2.86 (2.53–3.64)	
FT4 (ng/dl)		0.98 (0.86–1.29)	
TRAbs (IU/l)		5.31 (2.44–18.15)	
Total cholesterol (mg/dl)		200 (173–226)	
HDL (mg/dl)		53 (47.8–61.3)	
Triglycerides (mg/dl)		81.5 (60.8–115)	
LDLc (mg/dl)		123.3 (104.1–151.9)	
Glycaemia (mg/dl)		94 (87.8–102.3)	

Data are reported as number and percentage or median and interquartile range.

### Univariate and Binomial Logistic Regression Analysis according to CAS Evaluation

**Figure 1:**
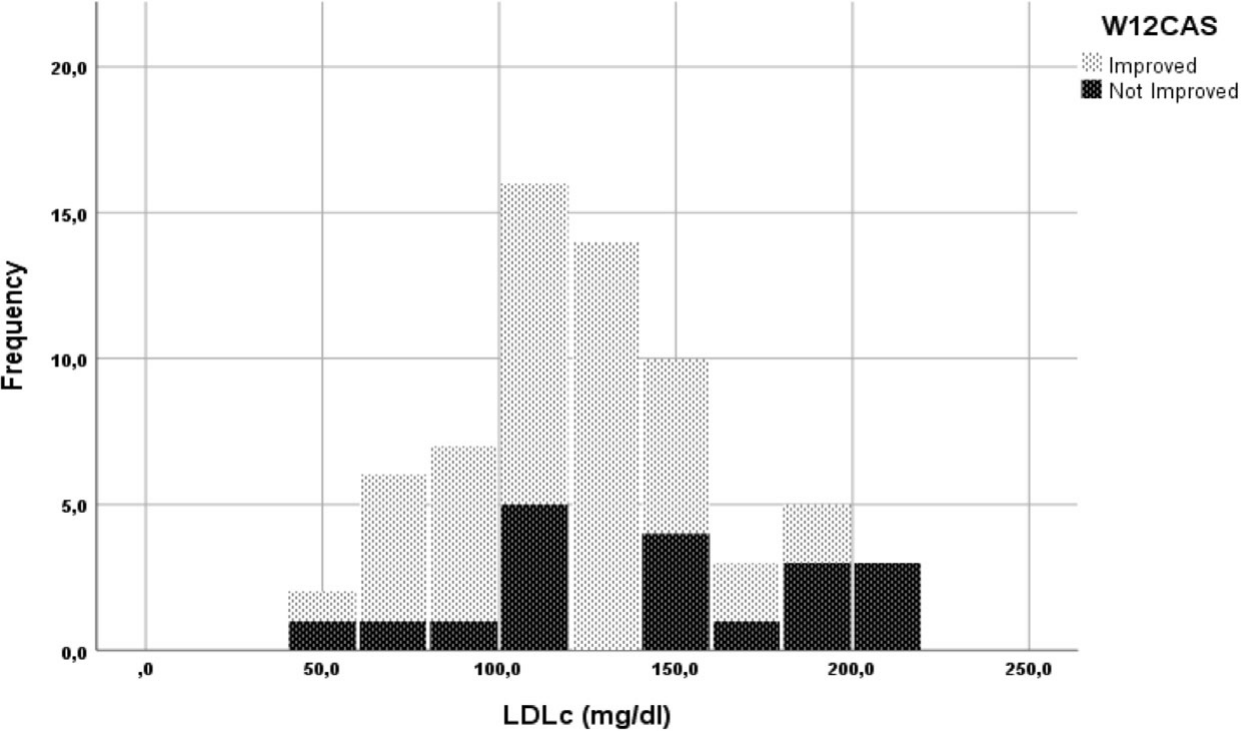
Stacked histogram of low-density lipoproteins cholesterol (LDLc) levels grouped for W12CAS categories.

On univariate analysis, the only two variables positively associated with GO improvement at 12W (I_W12CAS_) were serum levels of LDLc (r= -0.25, p=0.045) and early improvement at W6 (I_W6CAS_) (p<0.001) (**Figure 1**, **Table 2**). Notably, 90.6% patients classified as “improved” at W6 were also “improved” at W12. In contrast, 41.2% of patients ‘not improved’ at W6 (NI_W6CAS_) were classified as ‘improved’ at W12 (OR=13.714, CI 95% 3.61–52.095, p<0.001, I_W6CAS_ vs NI_W6CAS_).

**Table 2:** Univariate analysis for W12CAS outcome.

Variables	I_W12CAS_	NI_W12CAS_	p
Glycaemia (mg/dl)	93.5 (87.8–102)	95 (86.3–104.5)	0.885
Age*(years)	41.01	51.42	0.082
Females (%)	76.9	23.1	0.058
BMI (Kg/m^2^)	24.1 (21.7–27.9)	25.6 (21.3–28.6)	0.929
Smokers (%)	68.9	31.1	0.643
ivGC CD (mg/Kg)	50.4 (40.2–67.2)	60 (50.6–73.2)	0.131
Thyroid (ml)	18.6 (11.9–24.3)	18.1 (14.5–39.9)	0.546
CAS	3 (3–4)	3 (3–4)	0.927
TSH (mU/L)	0.05 (0–0.9)	0.05 (0.01–1)	0.661
FT3 (pg/ml)	2.87 (2.56–3.43)	2.69 (2.39–3.76)	0.699
FT4 (ng/dl)	0.98 (0.84–1.25)	1.05 (0.89–1.3)	0.975
TRAbs (IU/l)	4.6 (2.2–16.5)	8.4 (3.3–28)	0.156
Cholesterol*	31.67	39.89	0.094
HDL*	35.06	29.63	0.298
Triglycerides (mg/dl)	76.0 (58–112)	89.0 (74–131)	0.315
LDLc*	30.49	40.95	0.045
I-W6CAS (n, %)	48 (90.6)	5 (9.4)	<0.001

Data are expressed as number or percentage percentage, median and interquartile range or mean ranks as indicated (*); CD, cumulative dose.

We found a negative association between male gender and GO improvement at W12; however, this difference did not reach statistical significance (OR = 2.778, CI 95% 1.003–7.691, p=0.058). Similarly, higher total cholesterol and older age showed some association with the lack in GO improvement at W12, without reaching statistical significance (p=0.094 and p=0.08, respectively) There was no association between smoking habit and W12 outcome and no association with any of the other continuous variables considered (**Table 2**). Prior thyroidectomy was not a contributing factor as at 12W three out of six patients were classified as improved and three as not improved.

Variables identified at univariate analysis (LDLc and W6CAS) were then evaluated by binomial logistic regression to further ascertain their effects on the likelihood to affect GO improvement at W12. The continuous independent variable was found to be linearly related to the logit of the dependent variable. There were two standardized residuals with a value of -5.986 and -4.706 standard deviations, which were kept in the analysis. The logistic regression model was statistically significant, χ2(2) = 15.985, p < 0.001. The model explained 41.1% (Nagelkerke R^2^) (20) of the variance in W12CAS outcome and correctly classified 84.6% of cases. Sensitivity was 95.1%, specificity was 45.5%, positive predictive value was 86.7% and negative predictive value was 71.4%. Both predictor variables were statistically significant. I_W6CAS_ had 13 higher odds to be classified as I_W12CAS_ than NI_W6CAS_. Increasing LDLc was associated with a reduction in the likelihood of being classified as I_W12CAS_, since for each unit of LDLc reduction, the odds of improving at W12CAS increased by a factor of 1.03. **Table 3** reports the regression output of the final model. Based on the results of the binary logistic regression, the following model was constructed to predict the P(IW12CAS) (probability of improving at W12CAS) for individual patients [Eq. 2]:

(2) $$P\left(I_{W12CAS}\right)=\frac{1}{1+e^{-\left(2.95-0.025x₁+2.57x₂\right)}}$$

**Table 3:** Outputs of the binary logistic regression for the outcome variable W12CAS.

	B	SE	Wald	df	p	Odds Ratio	95% CI of Odds Ratio	
							Lower	Upper
**LDLc**	-0.025	0.013	4.025	1	0.045	0.975	0.951	0.999
**W6CAS**	2.570	0.863	8.858	1	0.003	13.065	2.405	70.976
**Constant**	2.950	1.707	2.987	1	0.084	19.099		

Final model built with covariates LDLc and W6CAS.

Where: *x*_1_ = LDLc (mg/dl); *x*_2_ = W6CAS outcome (*x*_2_ = 1 for I_W6CAS_; *x*_2_ = 0 for NI_W6CAS_). We tested the reliability of our model by performing several multivariate logistic regression analyses, with the aim of verifying that LDLc is an independent predictor for the W12CAS outcome. We also constructed an overfitted model constructed with 5 covariates (see supplementary data).

### Receiver Operating Characteristic Curve Analysis

**Figure 2:**
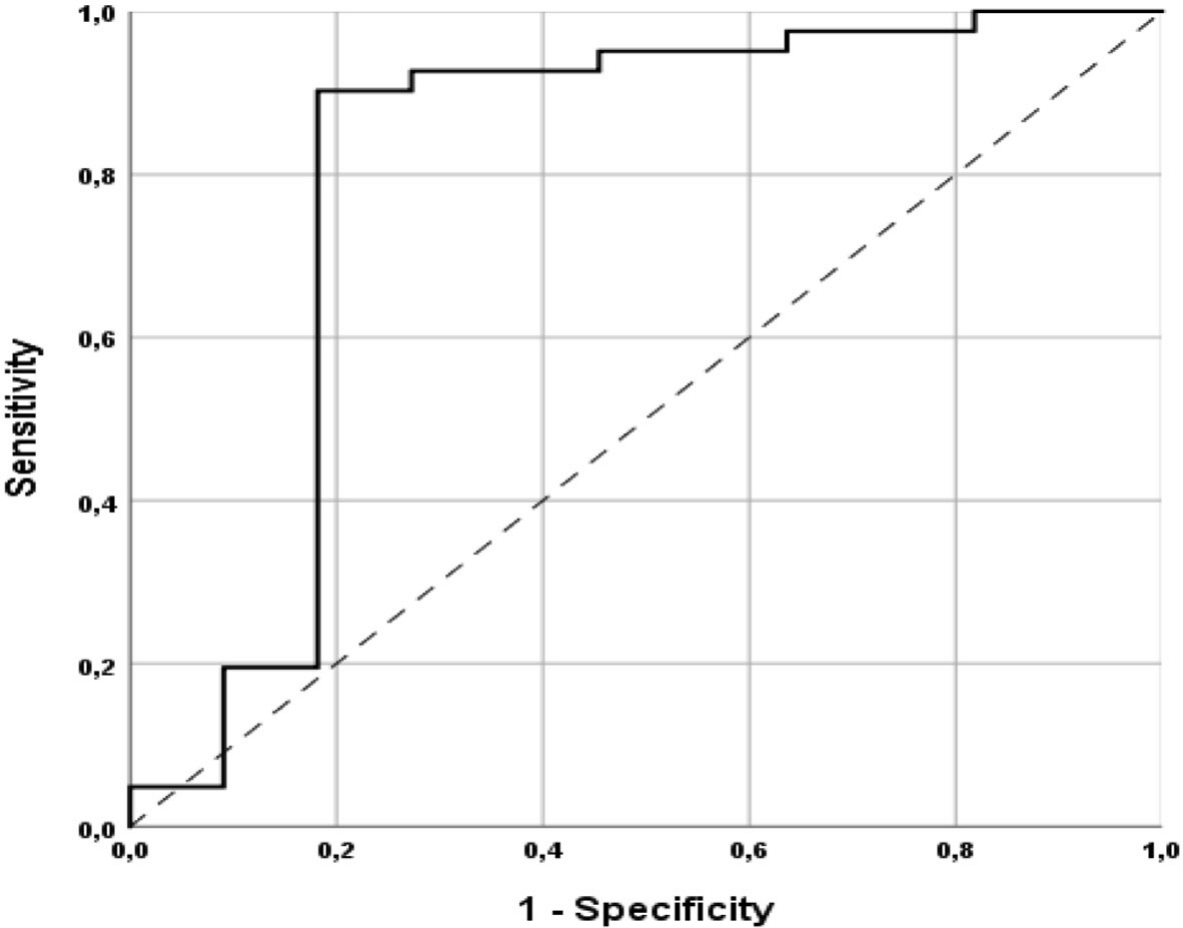
Receiver operating characteristic (ROC) curve of the predicted probability of improvement at W12CAS calculated with binomial logistic regression.

**Figure 3:**
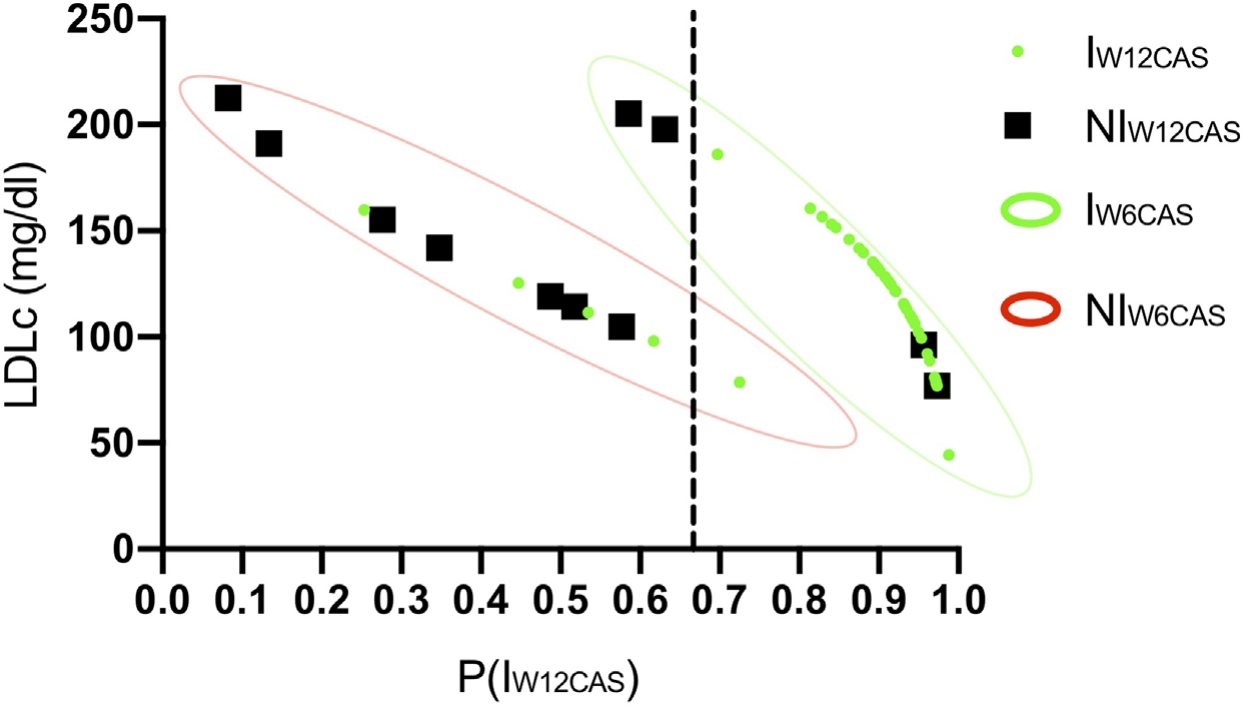
Suggested decision-making algorithm. The graphs are constructed on the basis of binomial logistic regression prediction using the Youden’s best cut-off P(I_W12CAS_) = 0.66. Each dot refers to a single patient; green dots refer to patients classified as Improved at W12CAS, while black dots refer to Not Improved Patients. The predicted probability of improvement at W12CAS [P(I_W12CAS_)] provided by regression analysis is positioned on the x axis; cut-off = 0.664 (dotted line). The continuous predictive variable included in the model (LDLc) is positioned on the Y axis. The categories of the dichotomous predictive variable (W6CAS outcome) are highlighted in green (I_W6CAS_) or red (NI_W6CAS_) contour lines.

The ROC curve processed using the regression P(I_W12CAS_) resulted in an excellent level of discrimination (0.805; 95% CI, 0.608–1.000) according to Hosmer et al. (23). (**Figure 2**). Youden’s test identified a P(I_W12CAS_) = 0.664 as best cut-off, with sensitivity 90.2%, specificity 81.8%, positive predictive value 94.9%, negative predictive value 69.2% and accuracy 88.5%. **Figure 3** highlights the impact that different P (I_W12CAS_) cut-offs have on the prediction performance of the model.

### W12CAS Binomial Logistic Regression Implementation

Almost every patient classified as “improved” at W6CAS had a P(I_W12CAS_) between 0.8 and 1.0, allowing a correct diagnosis in almost all of them. Within the I_W6CAS_ group the lower P(I_W12CAS_) (ranging from 0.59 to 0.7) was assigned to the patients with a very high LDLc and the Youden’s cut-off (unlike the standard cut-off P(I_W12CAS_) = 0.5) allowed to correctly predict their W12CAS outcome. Considering that the two independent variables (LDLc and W6CAS outcome) are not available at the same time, since the first one is obtained at baseline and the second one after a 6 weeks pulse therapy, we aimed at identifying a LDLc cut-off that would give a P(I_W12CAS_) <0.664 independently of W6CAS outcome; knowing this value may allow the physician to delay pulse therapy until this value is corrected. This LDLc cut-off was calculated using the binomial logistic regression model (Eq. 3) setting P(I_W12CAS_) < 0.664 (as indicated by Youden’s test):

(3) $$P\left(I_{W12CAS}\right)=\frac{1}{1+e^{-\left(2.95-0.025x₁+2.57x₂\right)}}<0.664\;$$

**Figure 4:**
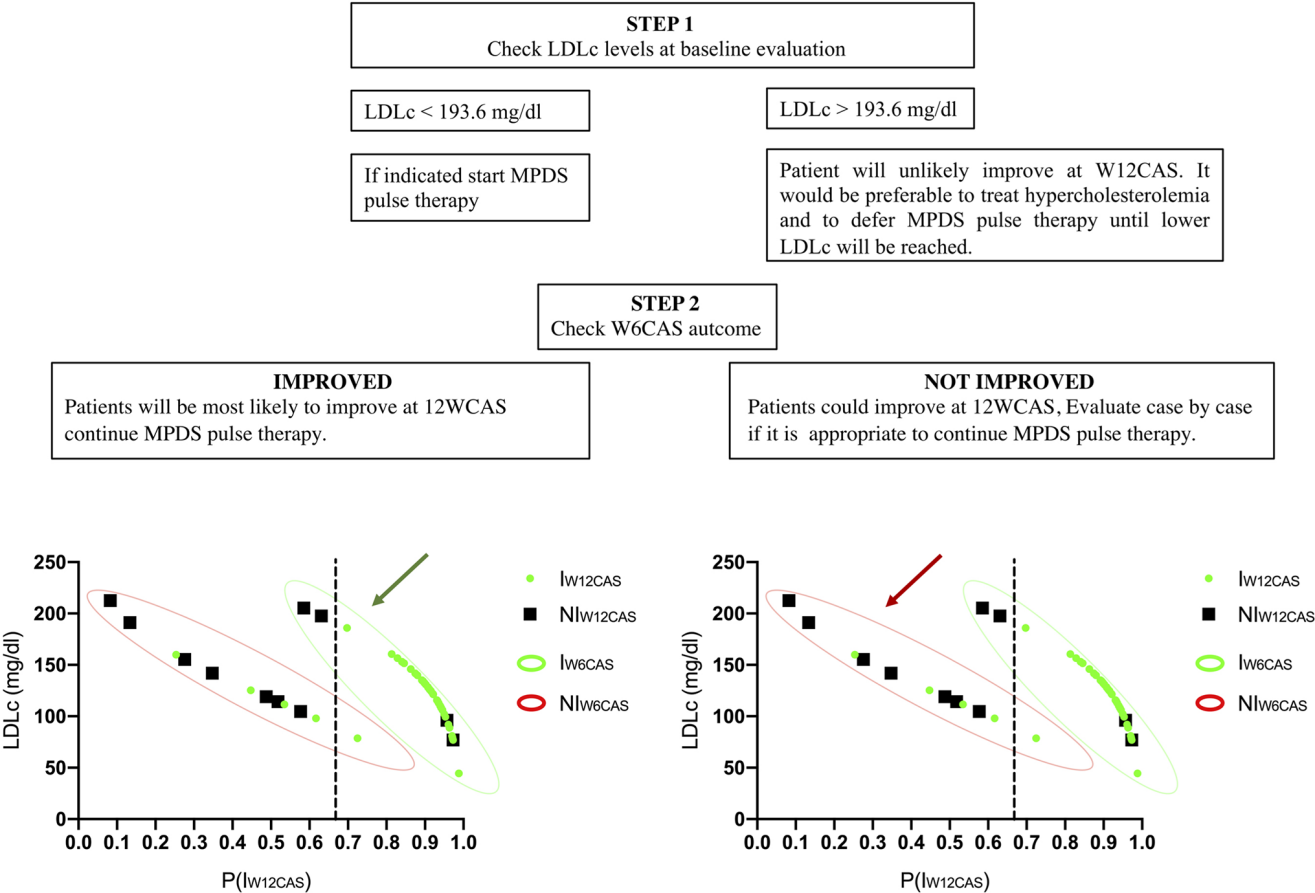
Steps of the decision-making algorithm.

The resulting inequality was solved for both x_2_ valid values (0 and 1) with the purpose to calculate which LDLc values (x_1_) would certainly give a P(I_W12CAS_) <0.664, independently from W6CAS outcome (x_2_). Calculations resulted in x_1_>193.6. According to this data, a patient showing a baseline LDLc >193.6 mg/dl can’t reach a P(I_W12CAS_) >0.664; in fact, in the best scenario (x_2_ = 1), he would reach a P(I_W12CAS_) value slightly lower than 0.664, while in the worst one (x_2_ = 0) its P(I_W12CAS_) should fall below 0.132. For W6CAS Improved patients, the outcome of the W6CAS itself is the most important predictor, with LDLc making a predominant contribution when it exceeds 190 mg/dL. Based on these results, a proposal for a decision algorithm was developed (**Figures 3** and **4**).

## Discussion

Herein we found that in patients with active GO addressed to pulse therapy with corticosteroids, baseline serum levels of LDLc and early clinical response at week 6 (W6CAS) were the strongest determinants of the clinical outcome at week 12 according to CAS evaluation (W12CAS). A previous study correlated serum total cholesterol and LDLc levels with the onset and severity of GO, but their impact on the GO response to corticosteroids has not been determined (9). In our study, we modeled the predicted probability P (I_W12CAS_) that each patient is classified as improved at W12CAS based on baseline serum levels of LDLc plus W6CAS outcome, showing that the LDLc level provides an independent contribution to the W6CAS data by overcoming the effect of W6CAS when it exceeds 190 mg/dl with a positive predictive value of 86.7% and a negative predictive value of 71.4%. Furthermore, the ROC curves indicated a sensitivity of 90.2% and a specificity of 81.8% in predicting a positive response of GO to corticosteroids at 12W.

Active GO is often an auto resolving condition, but several factors independently modulate its severity and ultimate clinical outcome. Although ivGC therapy is the most widely used medical treatment for active GO, its clinical efficacy is variable, and it is therefore important to have biomarkers that can predict its efficacy in the individual patient.

Few variables are known to influence the severity and outcome of GO. For example, smoking is a proven independent risk factor for the development and exacerbation of GO, and radioiodine (131-I) treatment of hyperthyroidism can also trigger GO and worsen its outcome. Furthermore, failure to achieve euthyroidism and persistence of high levels of TRAbs are negatively correlated with the clinical outcome of GO (24). Recently, a predictive risk score for the development of GO in patients with newly diagnosed Graves’ hyperthyroidism has been proposed (25). This score (PREDIGO) aims to estimate the odds ratio of GO exacerbation according to CAS based on serum levels of TSH and TRAbs, duration of hyperthyroidism and smoking habits. In our study, GO was moderate to severe and active in patients with stable thyroid function and with similar TRAbs levels between responders and non-responders, therefore the risk of GO exacerbation was similar across the study population. In addition to the aforementioned risk factors, GO occurrence and severity have been positively associated with diabetes mellitus (DM) (12, 26) and serum levels of total cholesterol and LDLc (9). These data are of interest since both DM and hypercholesterolemia induce a chronic inflammatory state that adds to GO immunological process and contributes to remodeling of the orbital tissues. Elevated LDLc levels have been hypothesized to increase the influx of free fatty acids into the liver causing the production of reactive oxygen radical species (ROS) and the secretion of interleukin-6 (IL-6). The increase in IL-6 activity within the orbital environment can promote greater secretion of insulin growth factor 1 (IGF-1) by orbital fibroblasts of GO patients and improve the proliferation of fibroblasts and the expansion of soft orbital tissues favoring IGF-1R - TSHR crosstalk (8, 27–29). These studies raise the need to investigate the impact of LDLc levels on the GO response to immunosuppressive therapy.

In our series of patients, none had previously received corticosteroids or retrobulbar radiation (30) and none had received radioiodine therapy to control hyperthyroidism. At week 6 after initiation of corticosteroid therapy, more than 90% of patients classified as responders maintained the same clinical rating at week 12. Corticosteroid responders at week 6 were significantly more likely than non-responders to have a positive outcome at week 12 (OR 13.7; p <0.001) confirming the previous observations (31). In our series, total cholesterol levels were higher in non-responders than in responders, but this difference did not reach statistical significance, while baseline LDLc levels were inversely related to GO clinical outcome at 12W. Other variables recognized as risk factor for GO presentation and/or exacerbation, such as cigarette smoking (24), male gender and age older than 60 yrs (32, 33) were not significantly predictive of GO response to ivGC at week 12. Although these findings may appear unexpected especially for smoking habit, the impact of smoking on the clinical response of GO to ivGC in a similar setting has not been previously investigated and deserves to be studied in prospective trials.

**Figure 5:**
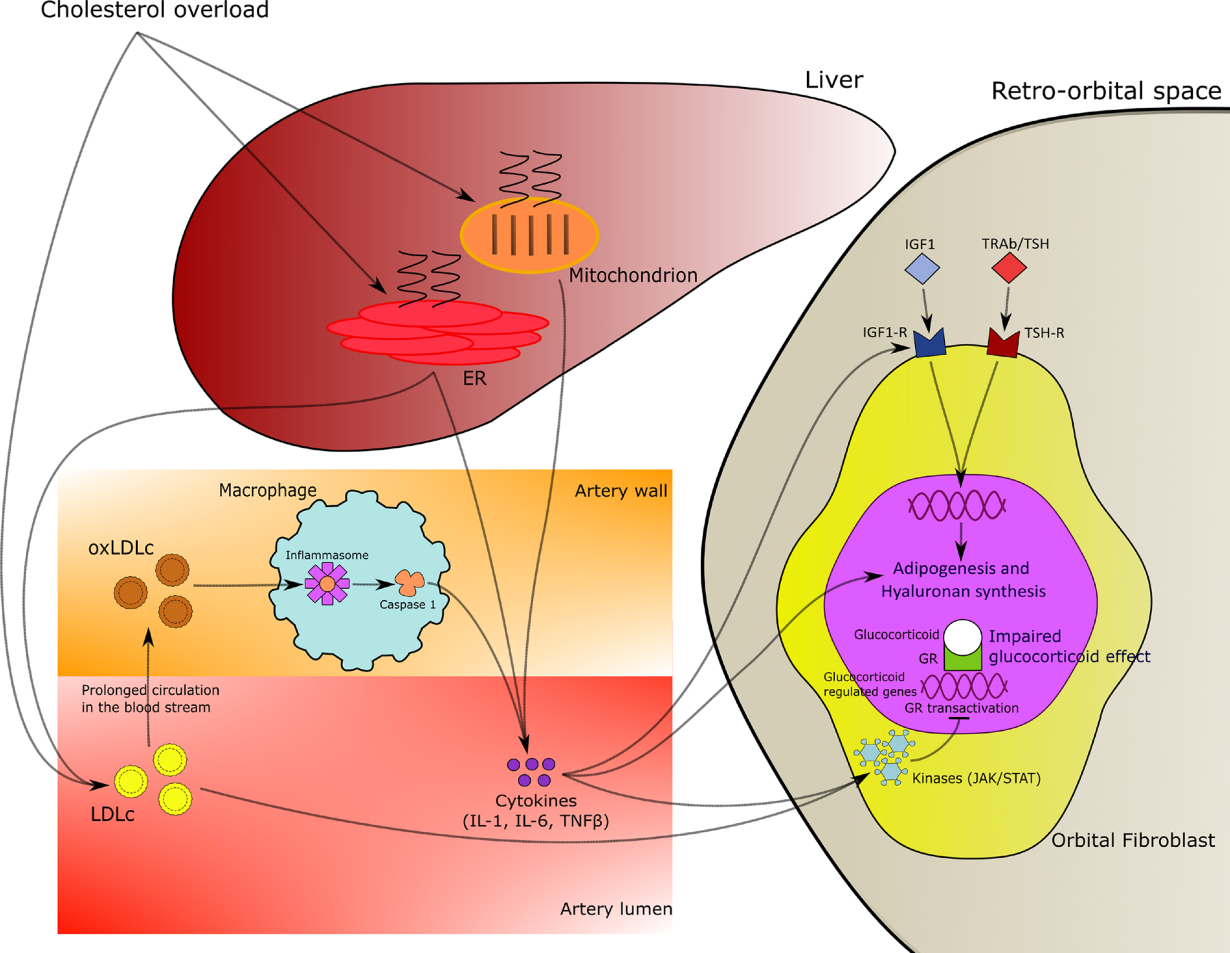
Mechanistic hypothesis of relationship of hypercholesterolemia with chronic inflammation and resistance to corticosteroids effects in patients with Graves’ ophthalmopathy (GO).

Our results are in agreement with the concept that hypercholesterolemia can promote chronic inflammation leading to resistance to the effects of corticosteroids characterized by inhibition of tissue macrophage functions, such as chemotaxis, phagocytosis, proliferation and antigen presentation (34). In particular, oxidized low-density lipoprotein (oxLDL) regulates the expression of dipeptidyl dipeptidase IV (DPP4) in macrophages leading to the increase of CD36 + cells which are representative of the inflammatory processes of atherosclerosis in obese and insulin resistant patients. Moreover, a direct effect of LDLc on transcriptional and translational activities of corticosteroids at the cellular levels has also been hypothesized (35, 36; **Figure 5**).

Our study has some limitations. Although all patients in our study were treated following a rigorous protocol, the study is retrospective, and the regression analysis was built on the basis of a small patient population. For these reasons, the reported algorithm is a suggestion that should be evaluated in a prospective context. However, our data indicate that patients with active GO may have a clinically limited response to ivGC therapy if LDLc levels are above 190 mg/dL.

## Conclusions

Early clinical response to ivGC therapy in patients with active GO is a strong determinant of clinical outcome at week 12 when assessed by CAS. Additionally, baseline serum LDLc levels are also an independent predictor of response to ivGC therapy, and LDLc levels above 190 mg/dL greatly reduce the likelihood of a positive clinical outcome at week 12. Based on our data, we suggest measuring baseline LDLc levels in all patients with active GO and strongly considering cholesterol-lowering treatment before referring these patients to ivGC therapy.

## Data Availability Statement

The raw data supporting the conclusions of this article will be made available by the authors, without undue reservation.

## Ethics Statement

The studies involving human participants were reviewed and approved by the Ethics Committee of Garibaldi Hospital—Catania. Written informed consent for participation was not required for this study in accordance with the national legislation and the institutional requirements.

## Author Contributions

RL conceptualized and designed the study. RL, AN, FG, MA, CR, FF, and DM collected the data. AN, RL, and FG performed the statistical analysis. RL and AN were in charge of the figures. RL, AB, FF, and AN wrote and revised the manuscript. All authors read and approved the final manuscript. All authors contributed to the article and approved the submitted version.

## Conflict of Interest

The authors declare that the research was conducted in the absence of any commercial or financial relationships that could be construed as a potential conflict of interest.

## Abbreviations

GD, Graves’ Disease; GO, Graves’ ophthalmopathy; ATD, anti-thyroid drugs; LDLc, low density lipoproteins cholesterol; PREDIGO, predictive score for the development or progression of Graves’ orbitopathy;, ivGC, intra-venous glucocorticoid; W6, week 6; W12, week 12; EuGoGo, The European Group on Graves’ Orbitopathy; FT3, triiodothyronine; FT4, thyroxine or tetraiodothyronine; TSH, thyroid stimulating hormone; TRAbs, thyrotropin (TSH)-receptor autoantibodies; RAI, radioiodine; ROC, receiver operator characteristics. CTEC, combined thyroid eyes clinic.

## References

[1] Bartalena L Baldeschi L Dickinson A Eckstein A Kendall-Taylor P Marcocci C. Consensus statement of the European Group on Graves’ orbitopathy (EUGOGO) on management of GO. Eur J Endocrinol (2008) 158:273–85. 10.1530/EJE-07-0666 18299459

[2] Soeters MR Van Zeijl CJJ Boelen A Kloos R Saeed P Vriesendorp TM. Optimal management of Graves orbitopathy a multidisciplinary approach. Neth J Med (2011) 69:302–8. 21934174

[3] Smith TJ Hegedüs L Douglas RS. Role of insulin-like growth factor-1 (IGF-1) pathway in the pathogenesis of Graves’ orbitopathy. Best Pract Res Clin Endocrinol Metab (2012) 26:291–302. 10.1016/j.beem.2011.10.002 22632366 PMC3712747

[4] Bartalena L Baldeschi L Boboridis K Eckstein A Kahaly GJ Marcocci C. The 2016 European Thyroid Association/European Group on Graves’ Orbitopathy Guidelines for the Management of Graves’ Orbitopathy. Eur Thyroid J (2016) 5:9–26. 10.1159/000443828 27099835 PMC4836120

[5] Xing L Ye L Zhu W Shen L Huang F Jiao Q. Smoking was associated with poor response to intravenous steroids therapy in Graves’ ophthalmopathy. Br J Ophthalmol (2015) 99:1686–91. 10.1136/bjophthalmol-2014-306463 26061160

[6] Shen L Huang F Ye L Zhu W Zhang X Wang S. Circulating microRNA predicts insensitivity to glucocorticoid therapy in Graves’ ophthalmopathy. Endocrine (2015) 49:445–56. 10.1007/s12020-014-0487-4 25588771

[7] Stein JD Childers D Gupta S Talwar N Nan B Lee BJ. Risk factors for developing thyroid-associated ophthalmopathy among individuals with graves disease. JAMA Ophthalmol (2015) 133:290–6. 10.1001/jamaophthalmol.2014.5103 PMC4495733 25502604

[8] Lanzolla G Vannucchi G Ionni I Campi I Sileo F Lazzaroni E. Cholesterol Serum Levels and Use of Statins in Graves’ Orbitopathy: A New Starting Point for the Therapy. Front Endocrinol (Lausanne) (2020) 10:1–8. 10.3389/fendo.2019.00933 PMC6987298 32038490

[9] Sabini E Mazzi B Profilo MA Mautone T Casini G Rocchi R. High Serum Cholesterol Is a Novel Risk Factor for Graves’ Orbitopathy: Results of a Cross-Sectional Study. Thyroid (2018) 28:386–94. 10.1089/thy.2017.0430 29336220

[10] Busnelli M Manzini S Froio A Vargiolu A Cerrito MG Smolenski RT. Diet induced mild hypercholesterolemia in pigs: Local and systemic inflammation, effects on vascular injury - Rescue by high-dose statin treatment. PloS One (2013) 8:1–15. 10.1371/journal.pone.0080588 PMC3829827 24260430

[11] Wang Y Patel A Douglas RS. Thyroid eye disease: How a novel therapy may change the treatment paradigm. Ther Clin Risk Manage (2019) 15:1305–18. 10.2147/TCRM.S193018 PMC6858302 31814726

[12] Mourits MP Prummel MF Wiersinga WM Koornneef L. Clinical activity score as a guide in the management of patients with Graves’ ophthalmopathy. Clin Endocrinol (Oxf) (1997) 47:9–14. 10.1046/j.1365-2265.1997.2331047.x 9302365

[13] Martin SS Giugliano RP Murphy SA Wasserman SM Stein EA Češka R. Comparison of low-density lipoprotein cholesterol assessment by Martin/Hopkins estimation, friedewald estimation, and preparative ultracentrifugation insights from the FOURIER trial. JAMA Cardiol (2018) 3:749–53. 10.1001/jamacardio.2018.1533 PMC6143070 29898218

[14] Van Isselt JW De Klerk JMH Van Rijk PP Van Gils APG Polman LJ Kamphuis C. Comparison of methods for thyroid volume estimation in patients with Graves’ disease. Eur J Nucl Med Mol Imaging (2003) 30:525–31. 10.1007/s00259-002-1101-1 12541136

[15] Rosenthal R. Meta-Analytic T Procedures Fob Social Research. Sage Publications (1991) 6. 10.4135/9781412984997

[16] Box GEP Tidwell PW. Transformation of the Independent Variables. Technometrics (1961) 4:531–50. 10.1080/00401706.1962.10490038

[17] Tabachnick BG Fidell LS. Using multivariate statistics. 6 th. California State University Northridge (2013).

[18] Menard S. Applied logistic regression analysis. Thousand Oaks: Sage University Paper. Sage Publications, Inc. (1995).

[19] Myers RH. Classical and modern regression with applications. 2nd ed. Boston: PWS Publishing Company (1990).

[20] Nagelkerke NJD. A note on a general definition of the coefficient of determination. Biometrika (1991) 78:691–2. 10.1093/biomet/78.3.691

[21] Hilbe JM. Logistic regression models. Boca Raton, Florida: Chapman and Hall/CRC (2009). 10.1201/9781420075779

[22] Youden WJ. Index for rating diagnostic tests. Cancer (1950) 3:32–5. 10.1002/1097-0142(1950)3:1<32::AID-CNCR2820030106>3.0.CO;2-3 15405679

[23] Hosmer DW Lemershow S Sturdiwant R. Applied logistic regression. New Jersey, US: John Willey & Sons Hoboken (2013). 10.1002/9781118548387

[24] Stan MN Bahn RS. 2010 Risk factors for development or deterioration of Graves’ ophthalmopathy. Thyroid (2010) 20:777–83. 10.1089/thy.2010.1634 PMC3357079 20578901

[25] Wiersinga W Žarković M Bartalena L Donati S Perros P Okosieme O. Predictive score for the development or progression of Graves’ orbitopathy in patients with newly diagnosed Graves’ hyperthyroidism. Eur J Endocrinol (2018) 178:635–43. 10.1530/EJE-18-0039 29650691

[26] Le Moli R Muscia V Tumminia A Frittitta L Buscema M Palermo F. Type 2 diabetic patients with Graves’ disease have more frequent and severe Graves’ orbitopathy. Nutr Metab Cardiovasc Dis (2015) 25:452–7. 10.1016/j.numecd.2015.01.003 25746910

[27] Lanzolla G Ricci D Nicolì F Sabini E Sframeli A Brancatella A. Putative protective role of autoantibodies against the insulin-like growth factor-1 receptor in Graves’ Disease: results of a pilot study. J Endocrinol Invest (2020) 43(12):1759–68. 10.1007/s40618-020-01341-2 32583374

[28] Marinò M Rotondo GD Ionni I Lanzolla G Sabini E Ricci D. Serum antibodies against the insulin-like growth factor- 1 receptor (IGF-1R) in Graves’ disease and Graves’ orbitopathy. J Endocrinol Invest (2019) 42:471–80. 10.1007/s40618-018-0943-8 30132285

[29] Douglas RS. Teprotumumab, an insulin-like growth factor-1 receptor antagonist antibody, in the treatment of active thyroid eye disease: a focus on proptosis *Eye (Lond)* (2019) 33:183–90. 10.1038/s41433-018-0321-y PMC6367366 30575804

[30] Boulanouar L Grunenwald S Imbert P Khalifa J Dekeister C Boutault F. Effect of orbital radiotherapy on the outcome of surgical orbital decompression for thyroid-associated orbitopathy (TAO): a retrospective study in 136 patients. Endocrine (2020) 67:605–12. 10.1007/s12020-019-02113-6 31646433

[31] Bartalena L Veronesi G Krassas GE Wiersinga WM Marcocci C Marinò M. Does early response to intravenous glucocorticoids predict the final outcome in patients with moderate-to-severe and active Graves’ orbitopathy? J Endocrinol Invest (2017) 40:547–53. 10.1007/s40618-017-0608-z 28176220

[32] Perros P Crombie AL Matthews JNS Kendall-Taylor P. Age and gender influence the severity of thyroid-associated ophthalmopathy: A study of 101 patients attending a combined thyroid-eye clinic. Clin Endocrinol (Oxf) (1993) 38:367–72. 10.1111/j.1365-2265.1993.tb00516.x 8319368

[33] Bereshchenko O Bruscoli S Riccardi C. Glucocorticoids, sex hormones, and immunity. Front Immunol (2018) 9:1–10. 10.3389/fimmu.2018.01332 29946321 PMC6006719

[34] Baschant U Tuckermann J. The role of the glucocorticoid receptor in inflammation and immunity. J Steroid Biochem Mol Biol (2010) 120:69–75. 10.1016/j.jsbmb.2010.03.058 20346397

[35] Rao X Zhao S Braunstein Z. Oxidized LDL upregulates macrophage DPP4 expression via TLR4/TRIF/CD36 pathways. EBioMedicine (2019) 41:50–61. 10.1016/j.ebiom.2019.01.065 30738832 PMC6441950

[36] Enuka Y Feldman ME Chowdhury A Srivastava S Lindzen M Sas-Chen A. Epigenetic mechanisms underlie the crosstalk between growth factors and a steroid hormone. Nucleic Acids Res (2017) 45:12681–99. 10.1093/nar/gkx865 PMC5727445 29036586

